# The Collaborative Governance Between Public and Private Companies to Address Climate Issues to Foster Environmental Performance: Do Environmental Innovation Resistance and Environmental Law Matter?

**DOI:** 10.3389/fpsyg.2022.936290

**Published:** 2022-07-08

**Authors:** Wei Sijing

**Affiliations:** Anyang Normal University Law School, Anyang, China

**Keywords:** collaborative governance, innovative methods, performance, availability of resources, environmental innovation resistance, environmental law, environmental performance

## Abstract

In the recent decade, the environmental problem is increasing significantly worldwide. With the decrease in environmental health, the environmental performance is decreasing continuously having adverse consequences for the societies. Therefore, to address the environmental problem in China, the current study examined the role of collaborative governance in environmental performance. Consequently, this study examined the relationship between collaborative governance, innovative methods, performance, availability of resources, environmental innovation resistance, environmental law, and environmental performance. Both the public and private companies of environmental protection working in China are considered. To address the objective of the study, a quantitative research approach is used along with the cross-sectional research design. A questionnaire survey is carried out among the public and private companies working in China for data collection. A total of 290 valid questionnaires were returned and used in data analysis. Partial Least Square-Structural Equation Modeling (PLS-SEM) is used for data analysis. Results of this study reported important findings which have a contribution to the literature and practice. Collaborative governance has major importance to enhance environmental performance. The collaboration between public and private companies has the potential to enhance environmental performance. It is found that an increase in collaborative governance can increase the innovative methods, performance, and availability of resources which can enhance environmental performance. The improvement in innovative methods, performance, and availability of resources can foster environmental performance. Furthermore, environmental innovation resistance can decrease environmental performance. Most significantly, environmental law is crucial to enhancing environmental performance. The better implementation of environmental law can enhance the environmental performance in China.

## Introduction

Environmental performance is one of the crucial issues globally (Ali et al., 2021; Rodríguez-Espíndola et al., 2022) because the environmental performance is decreasing significantly due to several reasons. Environmental performance below the satisfactory level led to generating several problems related to the health issues. Therefore, achievement of a satisfactory level of environmental health is a significant part of any society (Sharma et al., 2022). The promotion of human health and wellbeing requires environmental performance which is a major global challenge for the nations. Similar to the other nations, China is also facing the problem of environmental performance.

Although several measures are taken in China, the satisfactory level of environmental performance is not achieved. A number of companies are working to resolve the issues of environmental health (Du et al., 2020); however, these companies have not achieved a satisfactory level. The companies working on environmental performance are based on the public as well as private companies (Cui et al., 2020). Each company has its role to address the issues of environmental health and take various measures to protect the environment. Because environmental protection is a major challenge for companies (Liu et al., 2022), the highest level of environmental protection is not achieved in China. The environmental issues cause several other health issues to the general public which is one of the alarming situations for society. With the increase in business operations in China to serve a huge population, the pollution in the environment is increasing significantly. However, environmental law can promote environmental performance. Various laws to protect the environment can lead to higher environmental performance. Several policies are available in China based on environmental law, but the implementation of these laws is most important. However, environmental law is critical to protect the environment for the sustainability and betterment of the society. The collaborative governance is one of the important elements which can protect the environment in different ways.

Collaborative governance has the potential to influence the environment (Newig et al., 2018). Collaborative governance involves the government, community, and private sectors communicating with each other and working together to achieve more than any one sector could achieve on its own. Therefore, the collaboration between private companies as well as public companies can lead to better environmental performance. This collaboration between public and private companies is always based on environmental law and all the policies developed following environmental law. Most importantly, the implementation of environmental law in collaborative governance can mainstream the efforts to promote environmental performance. Similar to the current study, earlier studies also highlighted the important role of environmental law in environmental performance (Kumar et al., 2019).

Although a number of previous studies addressed environmental performance in China (Yu et al., 2021; Zhang et al., 2022), collaborative governance is not considered. For instance, Gu et al. (2020) highlighted the environmental performance in relation to the resource multiple-life-cycle recycling system. Miao et al. (2019) investigated environmental performance through the regulation effect of China's atmospheric pollutant emissions. Environmental performance is considered by Wu et al. (2018) by considering the transportation systems in China. Furthermore, Li and Ramanathan (2018) examined the relationship between environmental regulations and environmental performance. All these studies have considered environmental performance, but the role of collaborative governance is not considered by these studies. Apart from these studies, a larger number of previous studies on environmental performance have not considered collaborative governance in China. In addition, it is also observed that a significant part of the literature has covered environmental regulation; however, the moderating effect of environmental law is rarely addressed. Similarly, this study considered the environmental innovation resistance. The consideration of environmental innovation resistance is one of the unique aspects of this study. Although other studies highlighted innovation resistance (Joachim et al., 2018; Shin and Ahn, 2019), environmental innovation resistance is not considered.

Finally, from the aforementioned literature gap, this study derived a research question; what is the role of collaborative governance in environmental performance in the presence of environmental innovation resistance and environmental law? The objective of this study is to examine the role of collaborative governance in environmental performance in relation to environmental innovation resistance and environmental law. Thus, this study considered the relationship between collaborative governance, innovative methods, performance, availability of resources, environmental innovation resistance, environmental law, and environmental performance. This study has vital implications for the literature as well as practice. The theoretical contribution of this study leads to the important practical implications which have major insights for the practitioners to promote environmental performance through collaborative governance in China.

## Literature Review

### Collaborative Governance

According to Fernando (2019), management and planning are the primary factors necessary to prevail over rules of law. Hence, a group of people including representatives from government, public, and private sectors aimed to manage and plan cities, regions, and countries is called collaborative governance. Gostin et al. (2019) determined that due to insufficient, incomplete, and partial legal foundation for law and control, a set of problems prevails in societies. To promote a legal framework, collaborative governance plays a vital role. However, in many cases, collaborative governance in countries, such as China, itself lack prevailing the rules of law. Therefore, the enhancement of rules and law is one of the primary objectives of any collaborative governance. Moreover, Ma et al. (2018) determined that collaborative governance allows achieving what an individual sector cannot achieve on its own. In addition, with the help of collaborative governance, it becomes comparatively easy for a government to implement its policies and laws.

### Innovative Methods

Messerli et al. (2019) proposed that achievement of the desired result is always a challenge both for an individual and a government. The government takes steps to prevail laws and rules aiming to achieve their targets set for better governance. However, various methods such as innovative methods play a significant role in the achievement of the desired result. Chen et al. (2022) determined that innovative methods are helpful for the exploration and uncovering something new that encourages law implementation particularly based on equality. Furthermore, the implementation of laws and rules without any discrimination by a government or by the group of collaborative governance is always a difficult and challenging task. Innovative methods provide different effective ways of problems and solving them. Moreover, according to Sjödin et al. (2020) through innovative methods, complex problems are solved. In addition, societies' and people's awareness increase with the proper implementation of innovative methods.

### Performance

Eccles and Wigfield (2020) described that performance refers to the achievement of results for a specified task during the measurement period. Nonetheless, performance for collaborative governance demonstrates the amount of accomplishment, efficiency, and presentation. In addition, according to Hong and Ryu (2019), collaborative governance performance acts as a motive force that enables to understand complex issues involving government, public, and private sectors. Furthermore, the value of performance determines the value of teamwork and it also helps many stakeholders agree on solutions and tow work together. Another research conducted by Mojarad et al. (2018) determines that policy and lawmakers recognize and mark issues with the help of their performance. A performance essentially regulates the value of delivered actions and their effectiveness. Moreover, the effectiveness of collaborative governance is based on its performance. Therefore, performance of a collaborative governance plays a significant role.

### Availability of Resources

Grubler et al. (2018) described that availability of resources refers to the information about resources such as when resources are available, what conditions apply when resources are available, and how resources are usable for a determined project, goal, and target. There are some resources whose availability varies; hence, advance scheduling, appointment, or arrangement are required to make them available at a certain time. According to Newig et al. (2018), collaborative governance without allocation of resources and their availability normally remains unsuccessful to make a prominent performance. Hence, checking the availability of resources is necessary which plays an important role to increase the overall performance of collaborative governance. Desai and DeArmond (2021) determined that resources such as training rooms or consultants need advanced scheduling. In addition, the availability of resources is opposed to the destroyed and consumed resources. However, the availability of resources most of the time is dependent on the value of the budget for a project or task.

### Environmental Innovation Resistance

Khanra et al. (2021) concluded that traditional barriers often become the strongest barrier among all barriers, particularly, in the way of innovation. Barriers in the way of innovation are called innovation resistance. According to Huang et al. (2021), environmental innovation resistance refers to the barriers in the way of environmental innovation. These barriers include issues with the prevalence of rules of law, lack of purchase intention, value of the relationship among purchaser's demand, environment-friendly products, and environmental laws. There are certain types of barriers that limit environmental innovations. Changes focusing on the environment often face a variety of problems, sometimes these problems become the strongest obstructions in the way of environmental innovation. Sørensen and Torfing (2021) determined that collaborative governance and organizational implementations aimed at environmental innovation often fail without special attention and personal interest. Furthermore, environmental innovation resistance decreases the value of practical, political, and ambitious goals set by collaborative governance (Vedeld et al., 2021).

### Environmental Performance

Environmental performance refers to the method of numerically and quantifying marking the environmental achievement of a state's policies. Benzidia et al. (2021) stated that the state's policies play a vital role to achieve prominent environmental performance. Collaborative governance also has a prominent part in environmental performance. Collaborative governance is responsible to manage and to create a balance between emission, waste generation, and resource consumption. The performance of collaborative governance also depends upon the influence of products and business activities on the natural environment. In addition, according to Shah and Soomro (2021), manufacturing industries are widely consuming natural products, hence, these industries are one of the major reasons for unobtrusive environmental performance. Loizia et al. (2021) determined that during the COVID-19 outbreak, environmental performance decreases because manufacturing industries were rapidly consuming natural resources to meet their demand. Furthermore, Hanif et al. (2022) determined that advanced manufacturing technologies are also one of the major reasons to decrease environmental performance.

### Environmental Law

Karl and Karl (2022) determined that laws that provide care, conservation, preservation, and protection to the environment are called environmental laws. Regulatory regimes and collaborative governance focus administration, management, and control of specific natural resources such as fisheries, minerals, or forests. According to Boettcher (2022), regulation between the human and non-human world is necessary because it is important for the existence of both. International, national, and local entities enforce and enact environmental principles, directives, laws, policies, and regulations. Zhao et al. (2022) concluded that environmental law incorporates both law of pollution control and resources law, aiming to regulate human impact on the environment. Human health is the primary objective of environmental law because the manufacturing process, disposal of chemical material, and prohibiting importation bring risks to human health. Not only the stakeholders but it is also the responsibility of the government to implement the environmental law strongly with the help of government machinery to provide sustainability to the environment. Multinational organizations are working on the guidelines of corporate social responsibility to protect the environment (Dubey et al., 2019).

### Hypotheses Development

#### Collaborative Governance and Innovative Methods

Cronin et al. (2022) described that the involvement of multiple sectors means more innovation. Hence, the role of collaborative governance in China plays an important role in the implementation and exercise of innovative methods. A study based on multifunctional team, collaborative governance, and social innovation, conducted by Allal-Chérif et al. (2022), demonstrates that environmental laws prevail and sustainable ecological growth is observed in the areas where proper and effective exertion are applied aiming at innovative methods put into use. Results from previous literature are also evident that increase in the value of collaborative governance also increases the value of innovative methods. Hence, it is encapsulated as follows:

***H1:*** *Collaborative governance has a positive influence on innovative methods*.

#### Collaborative Governance and Performance

A good performance of multi-institutional governance involving various collaborators is always expected to boost the performance of government. Wang and Ran (2022) determined that collaborative governance deals with issues that arise due to entanglements, differences, and similarities that decrease the performance of collaborative governance. Furthermore, Mosley and Park (2022) also concluded that a great volume of collaborative governance increases its performance. In addition. Shi et al. (2022) also resolved that increased intention in environmental governance during urban-rural development process in Delt Region of River Yangtze increases the performance of collaborative governance of the region. Hence, it is encapsulated as follows:

***H2:*** *Collaborative governance has a positive influence on performance*.

#### Collaborative Governance and Availability of Resources

With a proper availability of leadership, support, and forum, collaborative governance is much more influential. According to Susha and Gil-Garcia (2019), collaborative governance plays a vital role to increase the availability of resources. Resources allocation and their discovery always are one of the challenging tasks for any government, however, collaborative governance has a significant positive influence in making them available. Furthermore, collaborative governance gathers the institutions into a forum aiming to have members collaborate to develop solutions, policies, and answers (Ulibarri, 2015). Hence, an increase in the value of collaborative governance results in an increase in the value of availability of resources. Therefore, it is encapsulated as follows:

***H3:*** *Collaborative governance has a positive influence on the availability of resources*.

#### Innovative Methods and Environmental Performance

A study examining the impact of eco-innovation on environmental performance conducted by Fernando and Wah (2017) determined that more practice of innovative methods results in maximizing the value of the green technology sector. Furthermore, Liao and Zhang (2020) determined that innovative methods adopted by responsible leadership have a positive influence on environmental performance. Results obtained from prior studies are also evident that large manufacturing especially in China practicing green innovation more frequently remains successful to increase their environmental performance (Bonamente and Aquino, 2019). Hence, it is concluded that an increase in the value of innovative methods promises an increase in the value of environmental performance. Therefore, it is encapsulated as follows:

***H4:*** *Innovative methods have a positive influence on environmental performance*.

#### Performance and Environmental Performance

According to Singh et al. (2020), improving environmental performance is one of the key responsibilities of any collaborative governance, especially in the countries that already have a greater network of manufacturing industries. China is the leading country that has a higher volume of their manufacturing, hence, environmental performance gets a significant role because manufacturing decreases environmental performance (Dubey et al., 2019). Furthermore, Chuang and Huang (2018) examining the effects of social responsibility and collaborative governance on environmental performance determined that higher value for collaborative governance and social responsibility cause higher value for environmental performance. Hence, results from prior study show that increases in the performance of collaborative governance also promises an increase in the performance in the value of environmental performance. Therefore, it is encapsulated as follows:

***H5:*** *Performance has a positive influence on environmental performance*.

#### Availability of Resources and Environmental Performance

A study examining relation through opportunity identification conducted by Memon et al. (2020) determined that the availability of resources extends confidence for vast choices and settlements. Several prior studies also concluded that the availability of resources has a positive significant impact on governance performance in many sectors such as the environment and green renewable energy sectors (Soares et al., 2017; Sharma et al., 2020). In addition, the availability of resources makes it comparatively easy to deal with uncertainty and disastrous conditions that usually decrease environmental performance. Hence, results obtained from previous literature indicate that increasing the value of available resources also increases the value of environmental performance. Therefore, it is encapsulated as follows:

***H6:*** *Availability of resources has a positive influence on environmental performance*.

#### Environmental Innovation Resistance and Environmental Performance

Singh et al. (2019) describing the role of environmental training determined that barriers to environmental innovation decrease environmental performance. A prior study is also evident that resistance to environmental innovation negatively affects sustainable environmental performance and continuedly reduces the managerial and environmental performances (Saudi et al., 2019). Furthermore, it is also obvious from the results of a prior study based on green innovation that increase in the value of environmental innovation resistance decreases the value of environmental performance. Hence, it is encapsulated as follows:

***H7:*** *Environmental innovation resistance has a negative influence on environmental performance*.

### Moderation Effects

Environmental law has positive effects on the relationship between innovative methods and environmental performance. More and a better practice of environmental laws increases the value of both the innovative methods and environmental performance. In addition, according to the results of a study focusing on green innovation and environmental law, human resources, and green transformational leadership remains successful to increase environmental performance by following environmental laws (Singh et al., 2020). Hence, it is encapsulated as follows:

***H8:*** *Environmental law moderates the relationship between innovative methods and environmental performance*.

Protection of air, soil, water, and land is necessary to preserve natural resources as well as to protect human health. Environmental laws are one of the major contributors that add significant meaning to the protection of human health and natural resources. Hence, the role of environmental laws is significantly positive for collaborative governance and environmental performance (Newig et al., 2018). Moreover, environmental law has positive effects on the relationship between collaborative performance by government and environmental performance. Hence, it is encapsulated as follows:

***H9:*** *Environmental law moderates the relationship between performance and environmental performance*.

A prior study evident from China conducted by Li and Ramanathan (2018) explored that environmental law helps in the acquisition of a cleaner environment that has a positive significant role in longer and healthier lives of people. Moreover, certain industries that relay on clean water and clean air, with the higher value of the implementation of environmental law, eventually result in more availability of resources. Hence, results from prior studies are evident that an increase in the value of environmental law also increases the value of moderation relationship between the availability of resources and environmental performance.

***H10:*** *Environmental law moderates the relationship between the availability of resources and environmental performance*.

Environmental innovation resistance has negative effects on the relationship between innovative methods and environmental performance. Past literature is also evident that increasing the value of environmental innovation resistance, decreases environmental performance and causes limitations that further cause repulsion for other innovative methods (Phan et al., 2018). Hence, it is encapsulated as follows:

***H11:*** *Environmental innovation resistance moderates the relationship between innovative methods and environmental performance*.

## Research Methodology

### Questionnaire Development and Pre-test

**Figure 1:**
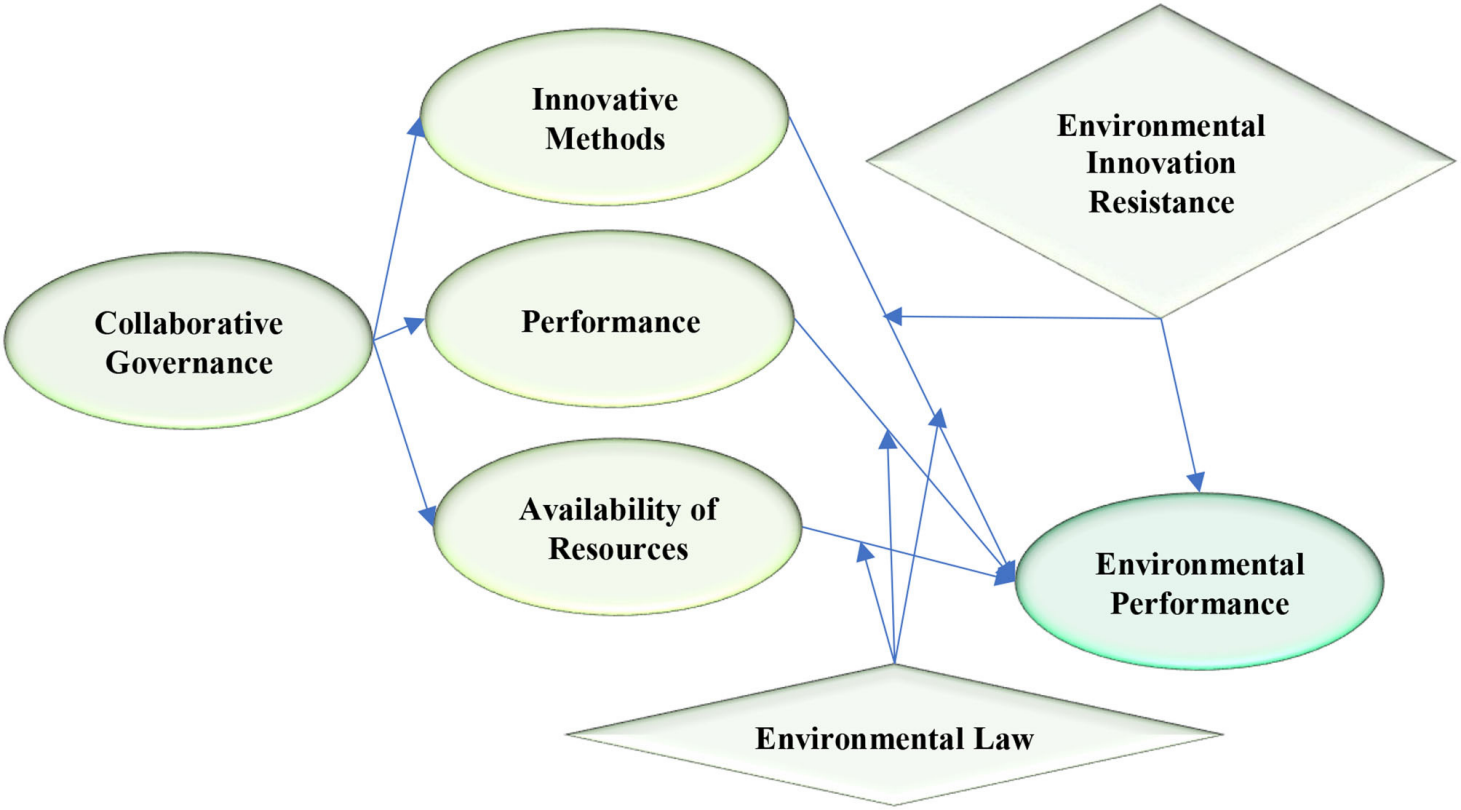
Research Model.

The current study adapted scale items from previous studies to measure the relationship between collaborative governance, innovative methods, performance, availability of resources, environmental innovation resistance, environmental law, and environmental performance. To measure collaborative governance, the current study adopted five scale items from Xing and Xing (2021). It is measured based on the collaboration of public and private companies to protect the environment. Four scale items are adapted from Hameed et al. (2018) to measure innovative methods. Four scale items are used to measure performance. These items are adapted from Chavez et al. (2013). In this study, performance is measured with the help of the operational performance of public and private companies working on environmental issues. Availability of resources is measured by using three scale items adapted from Brown (1996). Five scale items are used to measure environmental innovation resistance. These items are adapted from Liao and Zhang (2020). Furthermore, three items are used to measure environmental law which are adapted from Hernandez et al. (2010). Finally, environmental performance is measured by using five scale items adopted from Asiaei et al. (2022). All the measures are used to develop a questionnaire. These scale items are designed on Likert scale which is most suitable to collect data from respondents. To ensure the validity of the questionnaire, face validity and content validity is confirmed from the experts. I have developed this designed based on Research Model in Figure 1.

### Data Collection Procedure

The population of the study are the environmental protection companies. Both the public and private companies are considered in this study. The employees working in these companies were considered the respondents of the study. Furthermore, the companies working in China are considered for this study. Therefore, a questionnaire survey is carried out among the companies working in China. Most of the previous studies selected a 300 sample size. However, by following the literature, the current study selected 600 sample size. Thus, 600 questionnaires were distributed among the employees working in public and private companies in China. About 290 valid questionnaires were returned and used in data analysis. This study followed an online survey to collect data from the employees. Email addresses of the employees were gathered from the website of companies and questionnaires were distributed through email. The respondents were provided an introduction to the study and also all their queries related to the questionnaire were addressed. Further, the questionnaires were distributed with a simple data collection technique.

### Initial Data Screening

After the data collection, initial data screening is carried out. Initial data screening is carried out to fix the errors related to the missing value and outlier in the data. Both the missing values and outlier in the data have the potential to alter the results. While data screening all the issues related to the missing value, outlier in the data were resolved. Data statistics are given in Table 1. Normality of the data is also considered as shown in Table 1, however, Partial Least Square (PLS) is used in this study which is most suitable to analyze non-normal data (Hair et al., 2020). Moreover, this study identified common method bias (CMB) with the help of variance inflation factors (VIFs) engendered by the full collinearity test. It is found that all the variables have a VIF value below 3.3 which shows that there is no contamination of CMB in this study.

**Table 1:** Data statistics.

	**No**.	**Missing**	**Mean**	**Median**	**Min**	**Max**	**SD**	**Kurtosis**	**Skewness**
CG1	1	0	2.017	2	1	5	0.951	0.727	0.994
CG2	2	0	2.045	2	1	5	1.208	0.589	1.225
CG3	3	0	1.848	2	1	5	0.927	1.043	1.161
CG4	4	0	1.775	2	1	5	0.877	0.88	1.111
CG5	5	0	2.079	2	1	5	1.159	1.029	1.288
IM1	6	0	2.101	2	1	5	1.132	0.747	1.158
IM2	7	0	1.86	2	1	5	0.958	2.967	1.637
IM3	8	0	1.865	2	1	5	0.997	1.423	1.305
IM4	9	0	2.011	2	1	5	0.989	1.572	1.244
PER1	10	0	2.017	2	1	5	1.163	0.961	1.305
PER2	11	0	2.135	2	1	5	1.304	0.217	1.157
ER3	12	0	2.022	2	1	5	1.146	0.492	1.107
PER4	13	0	1.944	2	1	5	1.048	0.761	1.118
AR1	14	0	2.017	2	1	5	1.041	1.12	1.231
AR2	15	0	1.921	2	1	5	0.986	1.252	1.26
AR3	16	0	2.079	2	1	5	1.094	0.564	1.09
EIR1	17	0	1.955	2	1	5	1.101	0.534	1.16
EIR2	18	0	2.006	2	1	5	1.183	0.884	1.284
EIR3	19	0	1.815	2	1	5	0.98	2.127	1.465
EIR4	20	0	1.949	2	1	5	1.024	0.668	1.116
EIR5	21	0	2.208	2	1	5	1.1	−0.02	0.857
EL1	22	0	1.803	2	1	5	0.9	3.081	1.566
EL2	23	0	2.051	2	1	5	1.153	0.904	1.254
EL3	24	0	2.101	2	1	5	1.137	0.721	1.164
EP1	25	0	2.022	2	1	5	1.044	0.833	1.091
EP2	26	0	2.23	2	1	5	1.208	−0.015	0.938
EP3	27	0	2.09	2	1	5	1.133	0.206	1.013
EP4	28	0	2.309	2	1	5	1.245	−0.554	0.698
EP5	29	0	2.315	2	1	5	1.228	−0.41	0.811

*CG, Collaborative Governance; IM, Innovative Methods; PER, Performance; AR, Availability Of Resources; EIR, Environmental Innovation Resistance; EL, Environmental Law; EP, Environmental Performance*.

## Data Analysis

Partial Least Square-Structural Equation Modeling (PLS-SEM) is most suitable to analyze the primary data (Hair et al., 2020, 2021a,b). In social sciences, PLS-SEM is considered the most reliable data analysis technique. Therefore, the current study employed PLS-SEM to achieve the study objective. In the first step of PLS-SEM, this study confirmed the reliability and validity.

**Figure 2:**
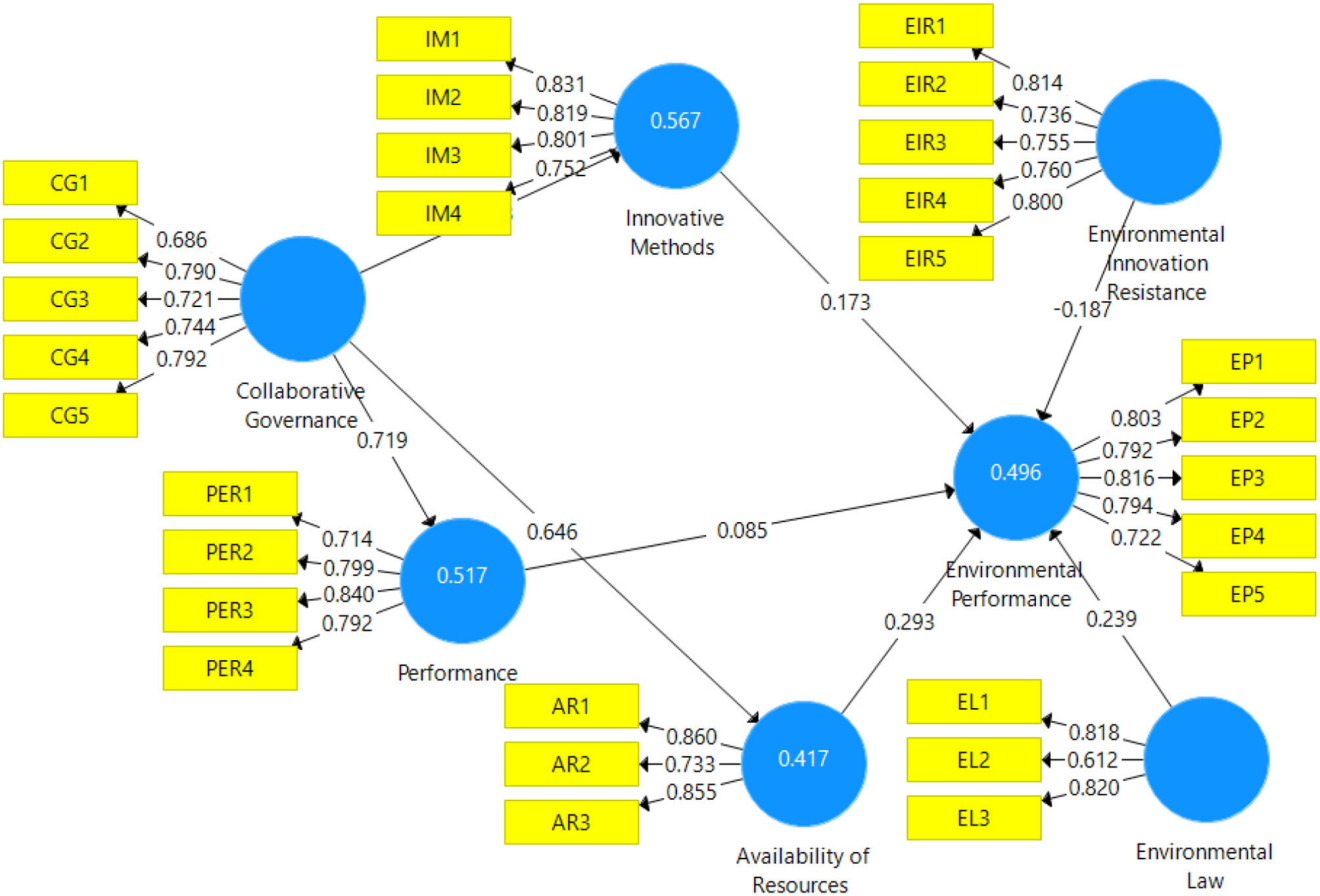
Measurement Model. CG, Collaborative Governance; IM, Innovative Methods; PER, Performance; AR, Availability of Resources; EIR, Environmental Innovation Resistance; EL, Environmental Law; EP, Environmental Performance.

The first step of PLS-SEM is based on the measurement model which is shown in Figure 2. To confirm the internal item's reliability, factor loading is considered. This study preferred 0.5 as the minimum threshold level of factor loadings to retain the scale items. All the factor loadings are given in Table 2 which shows that none of the items has factor loadings below 0.5. Thus, all the items are retained by the current study. Furthermore, composite reliability (CR) is considered which should be higher than 0.5. Results of the measurement model show that all the variables have CR above 0.7. In addition, to confirm the convergent validity, the average variance extracted (AVE) is considered which is above 0.5. Finally, in the measurement model, discriminant validity is achieved by using a heterotrait-monotrait ratio of correlations (HTMT)_0.9_ which is given in Table 3. All the values in Table 3 are <0.9 which confirmed the discriminant validity.

**Table 2:** Factor loadings and convergent validity.

**Variables**	**Items**	**Loadings**	**Alpha**	**CR**	**AVE**
Availability of resources	AR1	0.86	0.753	0.858	0.669
	AR2	0.733			
	AR3	0.855			
Collaborative governance	CG1	0.686	0.803	0.863	0.559
	CG2	0.79			
	CG3	0.721			
	CG4	0.744			
	CG5	0.792			
Environmental innovation resistance	EIR1	0.814	0.832	0.881	0.598
	EIR2	0.736			
	EIR3	0.755			
	EIR4	0.76			
	EIR5	0.8			
Environmental law	EL1	0.818	0.722	0.798	0.572
	EL2	0.612			
	EL3	0.82			
Environmental performance	EP1	0.803	0.845	0.89	0.618
	EP2	0.792			
	EP3	0.816			
	EP4	0.794			
	EP5	0.722			
Innovative methods	IM1	0.831	0.813	0.877	0.62
	IM2	0.819			
	IM3	0.801			
	IM4	0.752			
Performance	PER1	0.714	0.794	0.867	0.62
	PER2	0.799			
	PER3	0.84			
	PER4	0.792			

*CG, Collaborative Governance; IM, Innovative Methods; PER, Performance; AR, Availability Of Resources; EIR, Environmental Innovation Resistance; EL, Environmental Law; EP, Environmental Performance*.

**Table 3:** Discriminant validity.

	**Availability of resources**	**Collaborative governance**	**Environmental innovation resistance**	**Environmental law**	**Environmental performance**	**Innovative methods**	**Performance**
Availability of resources					
Collaborative governance	0.803						
Environmental innovation resistance	0.897	0.756					
Environmental law	0.703	0.853	0.701				
Environmental performance	0.773	0.643	0.721	0.825			
Innovative methods	0.833	0.827	0.678	0.786	0.689		
Performance	0.702	0.887	0.79	0.722	0.681	0.717	

**Figure 3:**
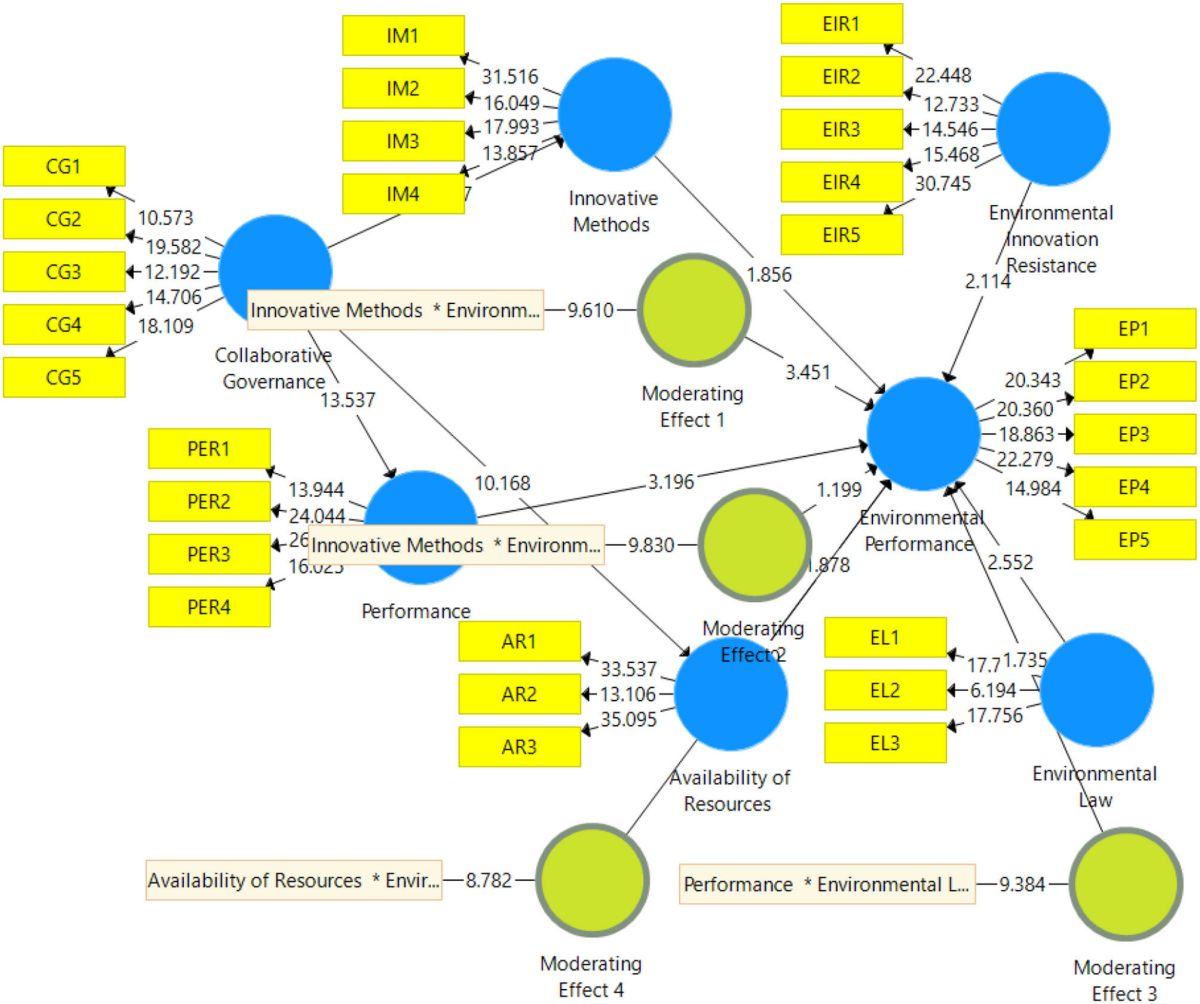
Structural Model. CG, Collaborative Governance; IM, Innovative Methods; PER, Performance; AR, Availability Of Resources; EIR, Environmental Innovation Resistance; EL, Environmental Law; EP, Environmental Performance.

**Figure 4:**
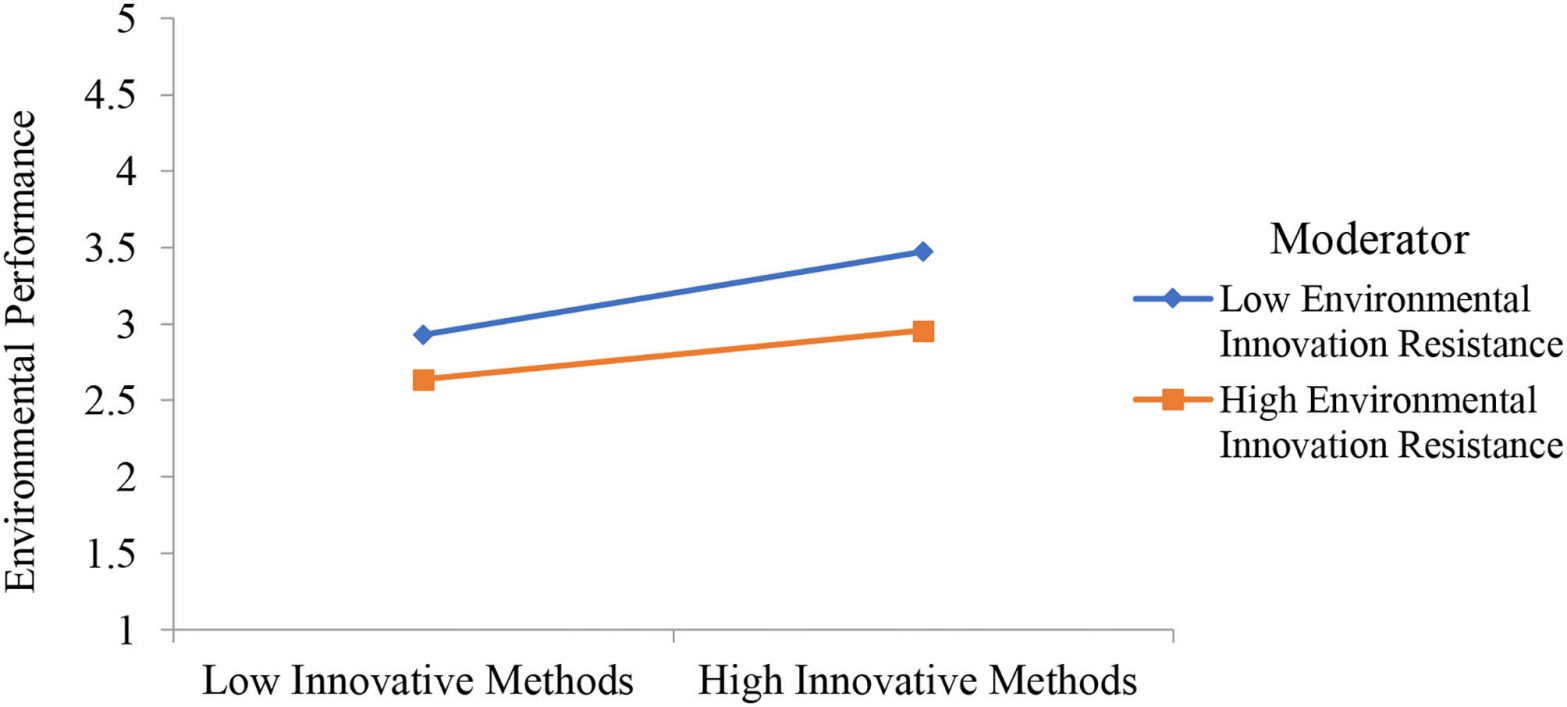
Moderation effect of environmental innovation resistance weakens the relationship between innovative methods and environmental performance.

**Figure 5:**
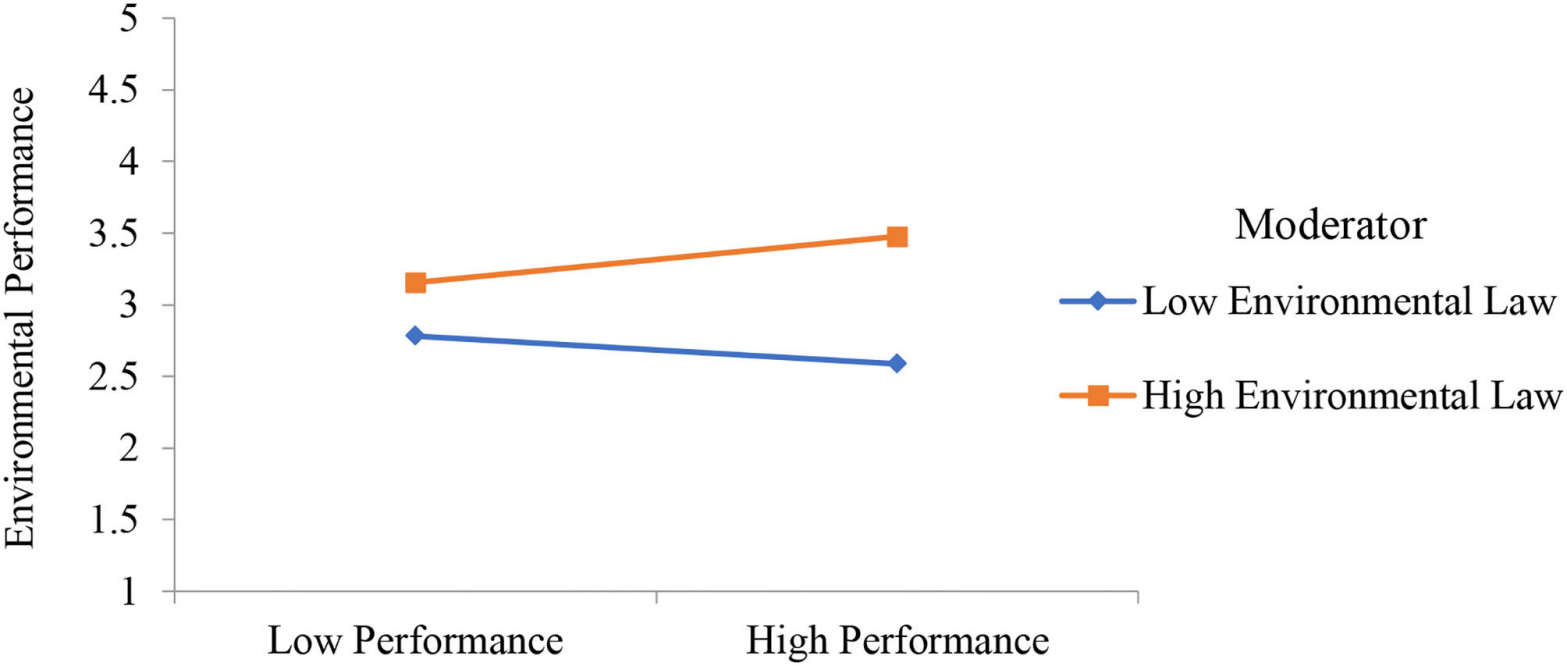
Moderation effect of environmental law strengthens the relationship between performance and environmental performance.

**Figure 6:**
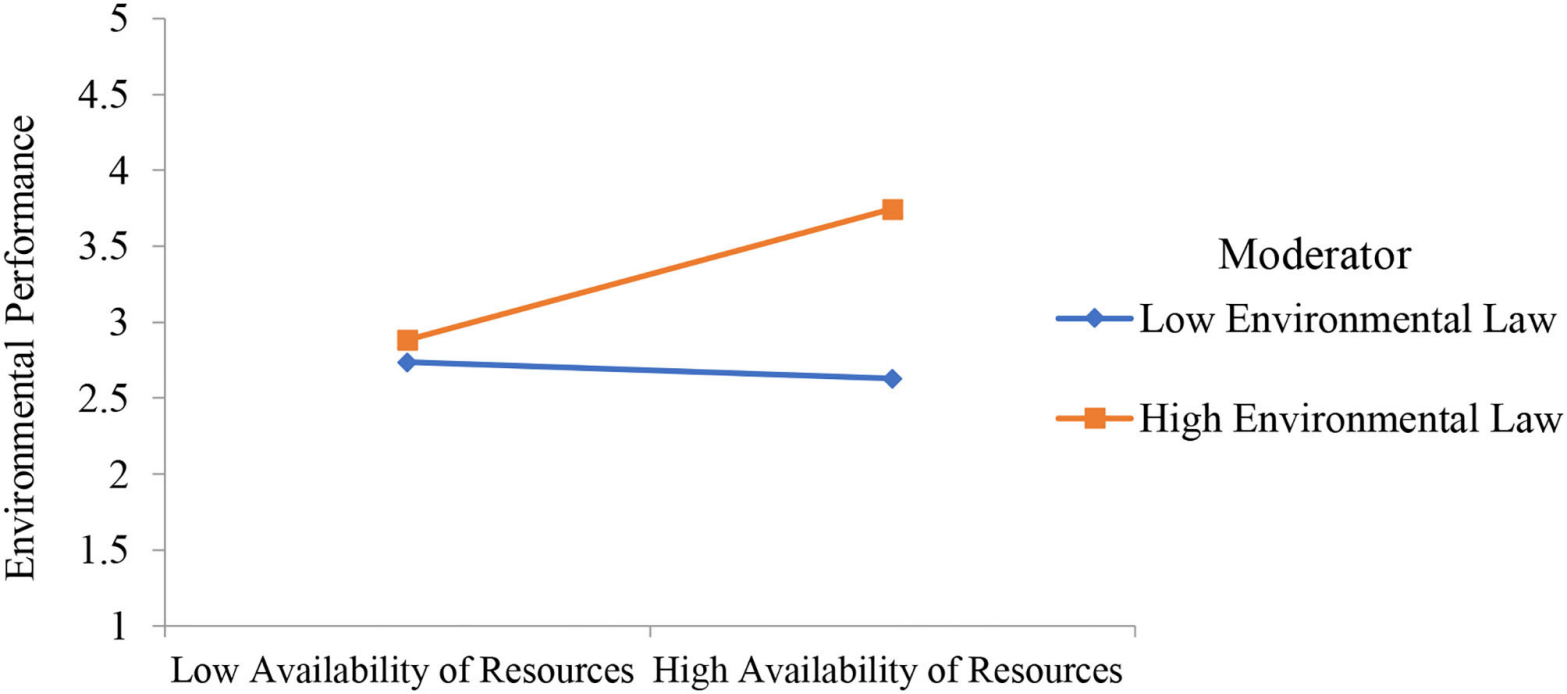
Moderation effect of environmental law strengthens the relationship between availability of resources and environmental performance.

The second part of PLS-SEM is based to examine the relationship between collaborative governance, innovative methods, performance, availability of resources, environmental innovation resistance, environmental law, and environmental performance. PLS measurement model is reported in Figure 3 and the results are reported in Table 4. Results show that collaborative governance has a positive effect on innovative methods, performance, and availability of resources. Furthermore, environmental innovation resistance has a negative effect on environmental performance and environmental law has a positive effect on environmental performance. Finally, the moderation effect is also given in Table 4 which shows that environmental innovation resistance as moderating variable is significant between innovative methods and environmental performance. Environmental law as moderating variable is significant between performance and environmental performance. It is also significant between availability of resources and environmental performance. All the significant moderation effects are presented in Figures 4–6. However, environmental law is not a moderating variable between innovative methods and environmental performance. The significance level is considered by examining the *t*-value of 1.64. Hypotheses having a *t*-value below 1.64 were considered as not supported.

**Table 4:** Direct effect results.

	**Original sample (O)**	**Sample mean (M)**	**Standard deviation (STDEV)**	**T statistics (|O/STDEV|)**	***P* values**
Availability of resources → Environmental performance	0.189	0.185	0.101	1.878	0.031
Collaborative governance → Availability of resources	0.646	0.644	0.064	10.168	0
Collaborative governance → Innovative methods	0.753	0.753	0.05	15.017	0
Collaborative governance → Performance	0.719	0.718	0.053	13.537	0
Environmental innovation resistance → Environmental performance	−0.215	−0.224	0.102	2.114	0.017
Environmental law → Environmental performance	0.316	0.342	0.124	2.552	0.006
Innovative methods → Environmental performance	0.216	0.219	0.117	1.856	0.032
Moderating effect 1 → Environmental performance	−0.056	−0.074	0.016	3.451	0
Moderating effect 2 → Environmental performance	−0.111	−0.107	0.092	1.199	0.116
Moderating effect 3 → Environmental performance	0.129	0.13	0.074	1.735	0.042
Moderating effect 4 → Environmental performance	0.243	0.244	0.103	2.35	0.01
Performance → Environmental performance	0.032	0.054	0.032	3.196	0

Finally, this study examined the variance explained in the dependent variable, environmental performance. It is observed with the help of the *r*-square value. Figure 2 shows that the *r*-square value is 0.496. It shows that all the variables such as collaborative governance, innovative methods, performance, availability of resources, environmental innovation resistance, and environmental law are expected to bring a 49.6% change in environmental performance.

## Discussion and Conclusion

To address the relationship between collaborative governance, innovative methods, performance, availability of resources, environmental innovation resistance, environmental law, and environmental performance, this study proposed 12 hypotheses which include eight direct hypotheses and four moderating effect hypotheses. The moderating role of environmental innovation resistance and environmental law is considered. The results of the study fulfilled the objective of the study.

Hypothesis 1 investigated the effect of collaborative governance on innovative methods. Results of this hypothesis highlighted that public and private companies' collaboration can invent innovative methods. This collaboration can help to promote innovative methods to enhance environmental performance. Similar to the current study, previous studies also show the important relationship between innovation and collaborative governance (Criado and Guevara-Gómez, 2021; van Gestel and Grotenbreg, 2021). Thus, the results of the current study are consistent with the previous studies. Hypothesis 2 highlighted that collaborative governance has a positive effect on performance. Better collaborative governance among the public and private environmental protection companies can enhance performance. Fanelli et al. (2019) also show a positive relationship between collaborative governance and performance. Similarly, Hypothesis 3 shows the relationship between collaborative governance and the availability of resources. Results show that an increase in collaborative governance can provide resources to the companies to fulfill major objective to enhance environmental performance. The integration between public and private companies can enhance the resources in China which can influence environmental performance. Furthermore, the results of the study proved that the role of innovative methods, performance, and availability of resources has a positive effect on environmental performance which is addressed in Hypotheses 4–6. In line with the current study, Seman et al. (2019) and Kraus et al. (2020) highlighted that innovation has a major role to enhance environmental performance.

Moreover, this study addressed the moderating role of environmental innovation resistance and environmental law. The direct effect of environmental innovation resistance on environmental performance is significant as shown in Hypothesis 7. These results show that environmental innovation resistance has a negative effect on environmental performance. The resistance of employees working in environmental safety companies is one of the major challenges while implementing innovative ideas. Therefore, the innovative resistance by the employees must be managed to implement new ideas. Hypothesis 8 shows the moderation effect of environmental law between innovative methods and environmental performance which is not supported. The moderation effect of environmental law strengthens the relationship between performance and environmental performance which is investigated through Hypothesis 9. Finally, Hypothesis 10 highlighted that the moderation effect of environmental law strengthens the relationship between the availability of resources and environmental performance. Hypothesis 11 shows the moderation effect of environmental innovation resistance between innovative methods and environmental performance which is significant. The moderation effect of environmental innovation resistance weakens the relationship between innovative methods and environmental performance. However, the data collection and analysis were not a simple task because there were different hurdles. Therefore, the respondents were accessed with easy to access approach.

Thus, it is concluded that collaborative governance has major importance to enhance environmental performance. The collaboration between public and private companies has the potential to enhance environmental performance. According to the current study, collaborative governance shows a positive role to enhance innovative methods, performance, and availability of resources which further enhances the environmental performance. In addition, resistance to innovation among public and private companies should be decreased to expedite environmental performance. Most importantly, environmental law is crucial in enhancing environmental performance. The companies should ensure the maximum implementation of environmental laws.

### Theoretical Implications

The current study addressed the important part of the literature and contributed significantly to the field of environmental performance and collaborative governance. First, environmental performance is addressed in relation to collaborative governance which is rarely addressed in previous studies. Although different studies addressed collaborative governance, it is not addressed in environmental performance. Second, the relationship between environmental performance and collaborative governance is not addressed in relation to the public and private companies in China. Third, this study addressed the moderating role of environmental innovation resistance which shows a negative role in environmental performance. This moderation effect is the first to be addressed in the literature. Fourth, this is the first study that considered the moderating role of environmental law. The role of environmental law between environmental performance and collaborative governance is most significant and is not addressed in the literature. Hence, the current study has a major contribution to the literature which leads to important theoretical implications.

### Practical Implications

Practically, the current study has major implications for public and private companies. The results of the study are helpful for the companies to promote environmental performance in China. The current study reported that collaborative governance has major importance for environmental performance. Therefore, the management of Chinese environmental protection companies should enhance collaborative governance. Furthermore, the results of the study recommended that the performance of low environmental performance in China can be managed with the help of environmental law. The implementation of environmental laws in China can enhance environmental performance. Furthermore, this study highlighted that resistance to innovation can decrease the environmental performance; therefore, management of environmental protection companies should decrease the resistance by encouraging the employees to foster innovative ideas.

## Data Availability Statement

The original contributions presented in the study are included in the article/supplementary material, further inquiries can be directed to the corresponding author/s.

## Ethics Statement

The studies involving human participants were reviewed and approved by the Lanzhou City University, China. The patients/participants provided their written informed consent to participate in this study. The study was conducted in accordance with the Declaration of Helsinki.

## Author Contributions

WS conceived, designed the concept, collected the data, and wrote the paper. The author read and agreed to the published version of the manuscript.

## Conflict of Interest

The author declares that the research was conducted in the absence of any commercial or financial relationships that could be construed as a potential conflict of interest.

## Publisher's Note

All claims expressed in this article are solely those of the authors and do not necessarily represent those of their affiliated organizations, or those of the publisher, the editors and the reviewers. Any product that may be evaluated in this article, or claim that may be made by its manufacturer, is not guaranteed or endorsed by the publisher.
